# Beyond Linear Measurements: A Multidimensional Framework for Evaluating Sexual Dimorphism in Human Postcanine Tooth Size

**DOI:** 10.3390/biomedicines14091937

**Published:** 2026-08-29

**Authors:** Srikant Natarajan, Junaid Ahmed, Shravan Shetty, Nidhin Philip Jose, Sharada Chowdappa, Sneha Kandala Sai

**Affiliations:** 1 Department of Oral Pathology and Microbiology, Manipal College of Dental Sciences Mangalore, Manipal Academy of Higher Education, Manipal 576104, India; srikant.n@manipal.edu; 2 Department of Oral Medicine and Radiology, Manipal College of Dental Sciences Mangalore, Manipal Academy of Higher Education, Manipal 576104, India; 3 Department of Orthodontics and Dentofacial Orthopaedics, Manipal College of Dental Sciences Mangalore, Manipal Academy of Higher Education, Manipal 576104, India; shravan.shetty@manipal.edu (S.S.); nidhin.philip@manipal.edu (N.P.J.); 4 Independent Researcher, Mangaluru 575003, India; mzsharada@rediffmail.com; 5 Independent Researcher, Hyderabad 500084, India; snejhaks975@gmail.com

**Keywords:** 3D geometry, centroid measurement, sexual dimorphism

## Abstract

**Background:** Sexual dimorphism in tooth size has been assessed on radiographs, dental casts, two-dimensional image analysis, and linear measurements are evaluated in two dimensions. These approaches evaluate isolated dimensions and may not adequately represent the three-dimensional complexity of tooth morphology. This study compared conventional odontometric measurements, centroid-based size assessments, and principal component-derived multidimensional size variables to determine whether overall tooth size exhibits sexual dimorphism. **Methodology**: A total of 120 dental casts (60 males and 60 females) were digitized using a laser surface scanner. Anatomical and geometric landmarks were identified on three-dimensional tooth models using 3D Slicer software. Mesiodistal, buccolingual, and diagonal dimensions were calculated from landmark coordinates, and two- and three-dimensional centroid sizes were derived. Principal component analysis was used to generate integrated multidimensional size variables. Sex differences were evaluated using independent Student’s *t*-tests, two one-sided tests (TOST) for equivalence, and random-effects meta-analysis. **Results**: Principal component analysis showed that conventional odontometric measurements and centroid sizes represented a common multidimensional size construct, explaining 79.1–91.3% of the variance across most tooth types. Centroid size demonstrated the highest component loadings, and principal component-derived variables correlated strongly with centroid size (r = 0.945–0.994; *p* < 0.001). No significant sex differences were observed for any principal component-derived size variable (*p* > 0.05). Equivalence testing and pooled meta-analysis demonstrated practical and statistical equivalence between males and females across principal component-derived variables, centroid sizes, and conventional odontometric measurements (all TOST *p* < 0.001). **Conclusions**: Conventional odontometric measurements, centroid sizes, and principal component-derived variables capture a common multidimensional construct of tooth size. Although isolated dimensions may differ, overall tooth size is practically equivalent between males and females.

## 1. Introduction

Human teeth are among the most morphologically stable structures of the craniofacial complex and have been extensively studied to understand biological variation, population differences, growth patterns, and sexual dimorphism. Variations in tooth dimensions have applications in dental anthropology, morphometric research, clinical dentistry, and forensic sciences [1,2]. Traditional odontometric research analyzed linear dimensions such as mesiodistal, buccolingual and diagonal measurements, with numerous studies reporting varying degrees of sexual dimorphism across different tooth types and populations [2,3,4,5].

The tooth has a complex three-dimensional structure. Linear measurements of a tooth provide a convenient method for evaluating tooth size, but they represent only isolated dimensions of a tooth. A tooth may exhibit differences along one dimension while simultaneously demonstrating compensatory changes along another dimension. Most odontometric research relies on measurements obtained from casts or radiographic images, which inherently reduce a complex three-dimensional structure to a two-dimensional plane. This flattening does not represent the true nature of the three-dimensional tooth structure and may further result in inaccuracies due to improper orientation or angulations during image acquisition. Consequently, statistically significant differences observed in individual measurements may not necessarily reflect meaningful differences in overall tooth size. This limitation raises an important methodological question regarding whether conventional odontometric measurements adequately represent the true multidimensional nature of tooth morphology.

Centroid size is a three-dimensional overall size measure derived from the spatial configuration of anatomical landmarks, calculated as the square root of the summed squared distances of all landmarks from their centroid. This provides a single quantitative representation of the overall dimensions of a biological structure. Conventional odontometric measurements evaluate isolated linear dimensions such as mesiodistal or buccolingual widths, whereas centroid size incorporates information in multiple dimensions from various anatomical landmarks simultaneously [6].

Principal component analysis (PCA) is a multivariate statistical technique that transforms a set of correlated variables into a smaller number of uncorrelated components that summarize the major sources of variation within the dataset. When applied to odontometric measurements, PCA integrates information from multiple dimensions and generates composite variables that represent underlying size-related patterns. Thus, whereas centroid size is a geometric measure directly derived from landmark coordinates, PCA is a statistical method that combines several measurements into an integrated multidimensional construct [6].

Recent advances involving analysis of the shape of the teeth, advanced statistical methods and morphometric analysis have introduced alternative approaches for evaluating biological size [7,8,9]. Geometric morphometry performed using landmark coordinates provides a geometric representation of size by incorporating information from multiple anatomical points simultaneously and produces the centroid area and volume in two and three dimensions respectively [10,11,12]. Similarly, principal component analysis enables the meaningful interpretation of size components by integrating multiple correlated measurements into a single multidimensional construct. The centroid size and use of principal component analysis enable researchers to evaluate overall tooth size which may be more meaningful than isolated linear dimensions in three-dimensional complex structures like the teeth [13].

Researchers obtain linear measurements using calipers, scales, and dividers or use image analysis software to derive measurements [2,4,14]. These methods may frequently lead to measurement errors due to the three-dimensional nature of teeth, as they fail to measure along the proper horizontal plane. These limitations can lead to discrepancies in recorded dimensions. The use of Cartesian coordinates not only measures the linear dimensions accurately but also provides researchers with an alternative methodology for representing tooth size in three dimensions. The coordinate geometry method uses the coordinate information of landmarks recorded along the x, y, and z axes and calculates linear distances using the Euclidean distance formula [15,16]. This approach allows us to measure specific dimensions not only in two dimensions, such as mesiodistal, buccolingual, and diagonal measurements, but also in three dimensions by deriving the centroid size, ensuring a comprehensive evaluation of tooth size.

Despite the growing application of geometric morphometric techniques in dental research, few studies have directly compared conventional odontometric measurements, centroid-based size assessments, and principal component-derived size constructs within the same population [17,18,19,20]. Furthermore, it remains unclear whether conclusions about sexual dimorphism differ depending on the method used to quantify tooth size. Understanding the relationship among these approaches is important for determining whether observed sex differences reflect genuine biological variation or arise from methodological differences in measurement.

Understanding the true extent of sexual variation in tooth size has broader implications for dental practice. Tooth dimensions influence digital workflow design, orthodontic diagnosis, restorative rehabilitation, prosthodontic planning, implant-supported restorations, and the development of artificial intelligence-based diagnostic models. Most existing approaches rely on isolated linear measurements to infer biological differences between males and females, despite the inherently three-dimensional nature of dental morphology. A comprehensive multidimensional evaluation may therefore provide a more biologically meaningful understanding of tooth size variation and help determine whether conventional linear odontometric differences translate into clinically relevant parameters [21,22].

Therefore, the present study aimed to compare conventional odontometric measurements, centroid size measurements, and principal component-derived multidimensional size variables across multiple tooth types. In addition, the study sought to evaluate the extent to which these approaches represent a common underlying size construct and whether they produce different conclusions regarding sexual dimorphism. We hypothesized that isolated linear dimensions may demonstrate significant sex-related differences; however, multidimensional approaches integrating multiple dimensions would reveal substantial overlap and statistical equivalence between sexes.

## 2. Materials and Methods

### 2.1. Sample Size Calculations

Sample size estimation was based on previously reported data on sexual dimorphism in mesiodistal dimensions of maxillary molars by Abe K et al. (1996) [23]. The reported mean ± standard deviation values of mesiodistal dimensions for males were 10.57 ± 0.52 mm (first molar) and 9.98 ± 0.58 mm (second molar), and for females they were 10.15 ± 0.55 mm (first molar) and 9.47 ± 0.60 mm (second molar). These values were used to calculate the required sample size for the comparison of means between two groups using the calculation of sample size for two means,

N = 2[[(Z_1−α/2_ + Z_1−β_)^2^]σ^2^]/d^2^ (1)

where d = clinically significant difference; σ = mean standard deviation. Using an alpha error of 1% (Z = 2.576), power of 90% (Z = 1.282), an average standard deviation of 0.535, and a clinically significant difference of 0.4 mm, the required sample size in each group was calculated as 54. The sample size was rounded to 60 per group to account for variability and improve robustness, resulting in a total of 120 individuals.

### 2.2. Study Data

**Figure 1:**
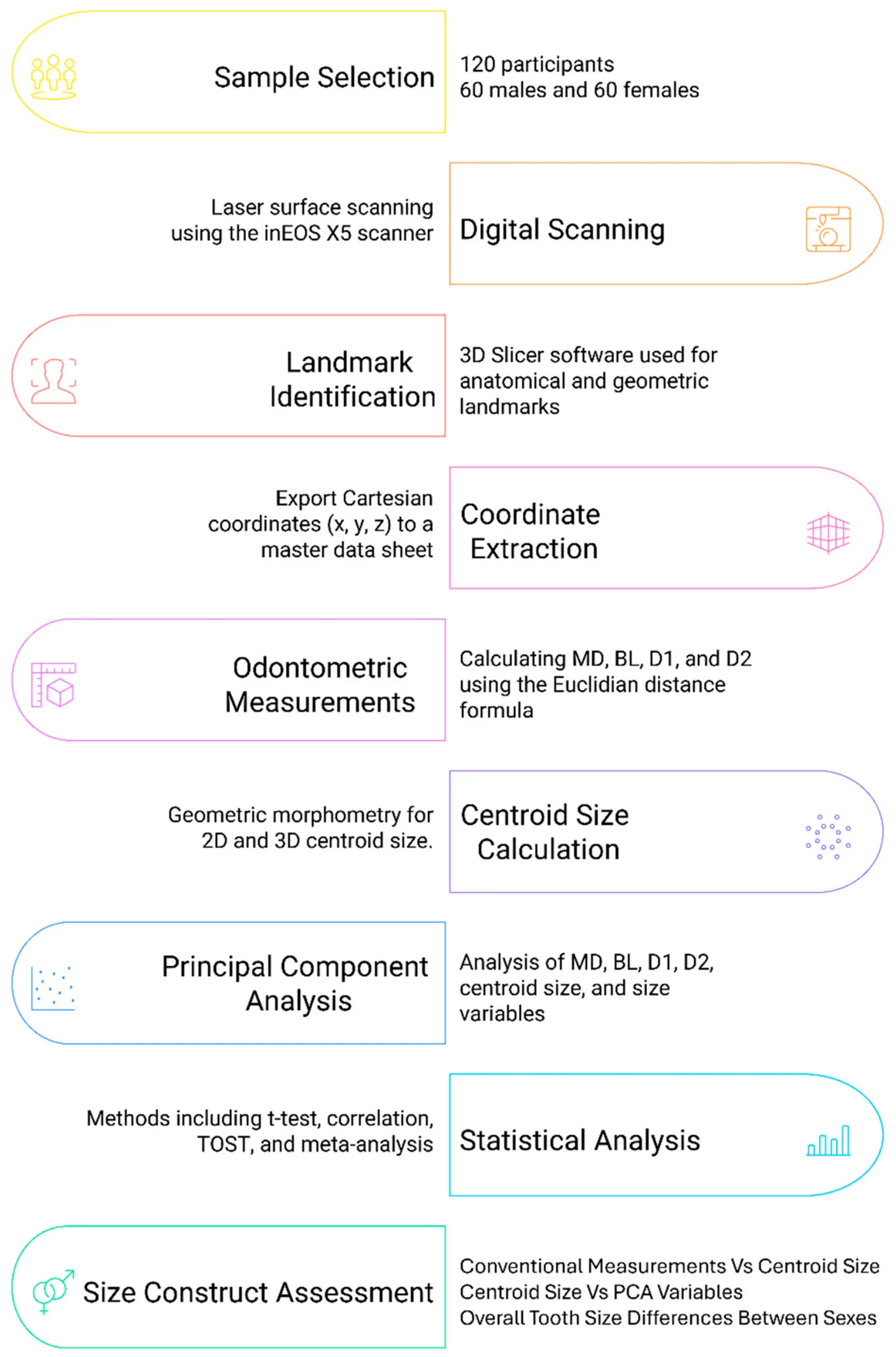
Workflow of the study showing sample selection, digital acquisition of three-dimensional tooth models, landmark-based measurements, derivation of centroid size variables, principal component analysis, and subsequent statistical analyses used to evaluate sexual dimorphism in postcanine tooth size.

In our study, we analyzed 120 dental casts of the maxilla and mandible, obtained from the Department of Orthodontics and Dentofacial Orthopedics, Manipal College of Dental Sciences, Mangalore. Clearance was obtained from the Institutional Ethics Committee (reference number 20018, dated 16 March 2020), and written informed consent was obtained from all participants. The department follows labeling dental casts with box numbers, and we included only non-identifiable demographic details (age and sex) with the collected coordinate data, ensuring that patient identities could not be traced directly or indirectly. As box numbers were used in place of personal identifiers and only age and sex were recorded, the dataset met anonymization standards appropriate for research use. The summary of the workflow is given in the flowchart (Figure 1).

### 2.3. Digital Model Acquisition and Landmark Identification

Impressions of the dental arches were made using Dentsply Aquasil Soft Putty and Kit (Dentsply, Noida, India) and casts were poured using Type 4 Extra Hard Dental Die Stone (Zhermack, Badia Polesine (RO), Italy). The casts were carefully dried and immediately scanned using a laser surface inEOS X5-Lab scanner (Dentsply Sirona, Noida, India) from the Department of Prosthodontics, Manipal College of Dental Sciences, Mangalore. Landmarks on the teeth were identified and marked using 3D Slicer software (version 4.10) (website: https://www.slicer.org, accessed on 5 January 2022) according to our previously published geometric morphometric protocols and landmark definitions for human postcanine dentition, which provide detailed landmark maps, anatomical definitions, and landmark acquisition procedures [24,25,26]. In brief, these landmarks were divided into points marked on the basis of anatomical and geometric evidence. Anatomical landmarks included cusp tips, ends of ridges, pits, and ends of grooves, while geometric landmarks were based on the maximum curvature or contour of the tooth surface, and junctions of two or three surfaces. The number of landmarks varied by tooth type, ranging from 19 in the maxillary second premolars to 32 in the mandibular molars. For linear measurements, the landmarks included the buccal and lingual crests of curvature for buccolingual dimension measurements in molars and premolars. For molars, the crest of curvature considered was the one associated with the mesiobuccal and mesiolingual ridges, because the teeth are wider buccolingually on the mesial side. The mesial and distal contact areas were assessed for both premolars and molars. Landmarks for the diagonal measurements consisted of the point angles, specifically the mesiobuccal, mesiolingual, distobuccal, and distolingual points. These landmarks were recorded in Cartesian coordinates in three dimensions (x,y,z) and used for subsequent measurements. These landmark points were measured in left and right teeth and extracted from Slicer3D software version 4.10, Boston, MA, USA and a masterchart was prepared in Microsoft Excel®. Further calculations were performed using the Excel spreadsheet.

### 2.4. Linear Measurements

Linear measurements were performed to calculate the mesiodistal, buccolingual, and diagonal dimensions of each tooth. To ensure true two-dimensional (2D) measurements, the z-coordinate was eliminated.

- Mesiodistal dimensions were measured as the distance between the most mesial and distal landmarks, typically corresponding to the contact points.
- Buccolingual dimensions were measured as the distance between the most buccal and lingual landmarks.
- Diagonal measurements included:
  1. Mesiobuccal-to-distolingual diagonal (diagonal 1 or D1);
  2. Mesiolingual-to-distobuccal diagonal (diagonal 2 or D2).

All these linear measurements were calculated using the 2D Euclidean distance formula after eliminating the z-coordinate:

d = sqrt[(x_2_ − x_1_)^2^ + (y_2_ − y_1_)^2^] (2)

### 2.5. Centroid Size Measurements

Centroid size was calculated using the Cartesian coordinates of all landmarks identified on each tooth. The centroid size is defined as the square root of the sum of squared distances of all landmarks from the centroid of the configuration (Bookstein, 1982) [27]. Centroid size represents a geometric measure of overall size and is defined as the square root of the summed squared distances of all landmarks from the centroid of the landmark configuration. Centroid sizes were calculated separately in two-dimensional (2D) and three-dimensional (3D) coordinate spaces. For 2D centroid size calculations, the z-coordinate was excluded, whereas all three coordinates (x, y, and z) were retained for 3D centroid size calculations.

To obtain a single representative value for each tooth type, the centroid sizes of the left and right homologous teeth were averaged for each individual. These values were subsequently used for sex comparisons and multivariate analyses.

### 2.6. Principal Component Analysis

Principal component analysis (PCA) was performed to investigate whether conventional odontometric dimensions and centroid size measurements represented a common underlying size construct. For each tooth type, the averaged mesiodistal, buccolingual, diagonal 1, diagonal 2, 2D centroid size, and 3D centroid size measurements were entered into the PCA. Components explaining meaningful proportions of variance were retained and interpreted based on their loading patterns. Component scores derived from the retained principal components were subsequently used to compare males and females. The percentage of variance explained by each component was recorded to evaluate the extent to which the variables represented a common multidimensional size construct.

### 2.7. Statistical Analysis

Statistical analyses were performed using Jamovi version 2.7.15 and MorphoJ software (version 1.08.01) [28,29]. Descriptive statistics, namely, measures of central tendency (mean and median) and dispersion (standard deviation and interquartile range), were calculated for all variables. Independent Student’s *t*-tests were used to compare mesiodistal, buccolingual, diagonal 1, diagonal 2, 2D centroid size, 3D centroid size, and principal component scores between the sexes. Effect sizes were reported as Cohen’s d with 95% confidence intervals. Pearson correlation analysis was performed to evaluate the association between principal component scores and 2D/3D centroid size measurements, to determine the extent to which centroid size and PCA-derived size variables represented the same underlying construct.

Two one-sided tests (TOST) were used for equivalence testing using equivalence bounds of ±0.5 Cohen’s d to evaluate practical equivalence between sexes. Statistical equivalence was concluded when both one-sided tests were significant (*p* < 0.05).

To compare the magnitude of sex differences across different measurement approaches, random-effects meta-analyses were performed separately for mesiodistal, buccolingual, diagonal 1, diagonal 2, principal component-derived size variables, 2D centroid size, and 3D centroid size for each tooth type. Pooled effect sizes, 95% confidence intervals, heterogeneity statistics (I^2^), and equivalence testing results were reported and further analyzed in second-order meta-analysis. For all analyses, a *p*-value less than 0.05 was considered statistically significant.

## 3. Results

### 3.1. Study Sample

The study sample comprised dental casts obtained from 120 individuals, including 60 males and 60 females. A total of 1858 postcanine teeth were available for analysis, and both left and right homologous teeth were included wherever present. Complete bilateral tooth records were available for the maxillary first premolars, maxillary second premolars, maxillary first molars, maxillary second molars, mandibular first premolars, mandibular first molars, and mandibular second molars in all 120 participants. The mandibular second premolars were further classified according to occlusal morphology into two-cusp and three-cusp variants. The two-cusp mandibular second premolar group comprised 20 males and 29 females (n = 49), whereas the three-cusp mandibular second premolar group comprised 47 males and 64 females (n = 111). Sample sizes therefore varied slightly according to tooth type and morphological variant.

### 3.2. Principal Component Analysis

The principal component analysis demonstrated that the conventional odontometric measurements (mesiodistal, buccolingual, diagonal 1, and diagonal 2) and centroid size measurements represented a common underlying size construct for all tooth types (Table 1). The component loadings showed that the centroid size measurements consistently exhibited the highest loadings, indicating the strongest contribution to the size construct. The 2D centroid size demonstrated loadings ranging from 0.967 to 0.994, while the 3D centroid size demonstrated loadings ranging from 0.945 to 0.992. In contrast, the conventional odontometric measurements showed lower loading ranges: mesiodistal (0.752–0.940), buccolingual (0.755–0.925), diagonal 1 (0.843–0.950), and diagonal 2 (0.847–0.960). For all teeth except the maxillary first molar, a single principal component adequately represented overall tooth size, explaining between 79.1% and 91.3% of the total variance. The highest variance explained was observed for the mandibular second premolar (two-cusp type) (91.3%) and the least was in the mandibular first premolar (79.1%) (Table 1).

Among the conventional odontometric measurements, diagonal measurements generally demonstrated higher loadings than mesiodistal and buccolingual dimensions. The maxillary first molar demonstrated a distinct loading pattern. Two principal components were retained, together explaining 82.2% of the total variance. The conventional odontometric measurements loaded predominantly on PC1, which explained 48.1% of the variance, whereas the centroid size variables loaded predominantly on PC2, which explained an additional 34.1% of the variance. Despite this separation, both components represented substantial proportions of the overall variation in tooth size.

Overall, the consistently higher loadings observed for both 2D and 3D centroid size measurements suggest that centroid-based approaches capture the common size variation of the tooth more effectively than any individual linear odontometric dimension.

### 3.3. Correlation Between Principal Component-Derived Size Variables and Centroid Size Measurements

The correlations between the principal component-derived size variables and centroid size measurements are presented in Table 2. Strong, significant positive correlations (ranging from 0.945 to 0.994) were observed between the principal component scores and centroid sizes across all tooth types (*p* < 0.001).

Regarding correlation with 2D centroid size, the strongest correlation was observed with the mandibular second premolar (two-cusp type) (r = 0.994; *p* < 0.001), followed by the mandibular second premolar (three-cusp type) (r = 0.992; *p* < 0.001). Similarly, very high correlations were observed for the maxillary second premolar (r = 0.989; *p* < 0.001), mandibular first molar (r = 0.985; *p* < 0.001), mandibular second molar (r = 0.985; *p* < 0.001), maxillary second molar (r = 0.985; *p* < 0.001), maxillary first premolar (r = 0.984; *p* < 0.001), mandibular first premolar (r = 0.983; *p* < 0.001), and maxillary first molar (r = 0.967; *p* < 0.001).

For the 3D centroid size, the strongest correlations were observed for the mandibular second premolar (two-cusp type) (r = 0.992; *p* < 0.001), maxillary second premolar (r = 0.986; *p* < 0.001), mandibular second premolar (three-cusp type) (r = 0.986; *p* < 0.001), and mandibular first molar (r = 0.985; *p* < 0.001). Correlations remained consistently high for the maxillary first premolar (r = 0.973; *p* < 0.001), mandibular second molar (r = 0.972; *p* < 0.001), mandibular first premolar (r = 0.969; *p* < 0.001), maxillary first molar (r = 0.963; *p* < 0.001), and maxillary second molar (r = 0.945; *p* < 0.001).

These findings indicate that the principal component-derived size variables and centroid size measurements represent a common underlying size construct. The uniformly high correlation coefficients suggest that both approaches capture overall tooth size in a highly comparable manner despite being derived using different computational methodologies (Table 3).

Comparison of principal component-derived size variables between males and females demonstrated no statistically significant sex differences for any tooth type (all *p* > 0.05). The effect sizes were uniformly small, ranging from 0.012 to 0.300 in magnitude. The smallest effect was observed for the mandibular second premolar (three-cusp type) (Cohen’s d = 0.012; *p* = 0.950), whereas the largest effect was observed for the mandibular second molar (Cohen’s d = −0.300; *p* = 0.103). For the maxillary first molar, centroid measurements loaded on PC2 rather than PC1; however, comparison of PC2 scores between sexes also revealed no significant difference (Cohen’s d = −0.289; *p* = 0.116). Collectively, these findings indicate substantial overlap in multidimensional tooth size between males and females across all tooth types.

### 3.4. Equivalence Testing of Principal Component-Derived Size Variables

The results of the two one-sided tests (TOST) equivalence analysis are presented in Table 4. Conventional null-hypothesis significance testing (NHST) revealed no statistically significant sex differences in the principal component-derived size variables for any of the evaluated tooth types (all *p* > 0.05). However, equivalence testing provided additional evidence regarding the practical similarity of tooth size between males and females.

Statistical equivalence was demonstrated for the maxillary first premolar, maxillary second premolar, mandibular first premolar, and mandibular second premolar (three-cusp type), as both lower and upper one-sided tests were statistically significant (all *p* < 0.05) indicating equivalence.

The maxillary first molar (PC1 and PC2), maxillary second molar, mandibular first molar, and mandibular second molar failed to demonstrate equivalence because the lower-bound test remained non-significant (*p* > 0.05), despite significant upper-bound tests. Similarly, the mandibular second premolar (two-cusp type) failed to demonstrate equivalence because the upper-bound test was non-significant (*p* = 0.085). Importantly, none of these tooth types demonstrated statistically significant sex differences in the NHST analysis, indicating that the results were inconclusive rather than suggestive of sexual dimorphism.

Overall, four of the nine evaluated tooth categories demonstrated statistical equivalence between males and females, whereas the remaining five categories showed insufficient evidence to establish equivalence within the predefined equivalence bounds. Notably, no tooth type demonstrated significant sex differences in the integrated principal component-derived size variables (Table 4).

### 3.5. Meta-Analysis of Sex Differences Across Measurement Methods

**Figure 2:**
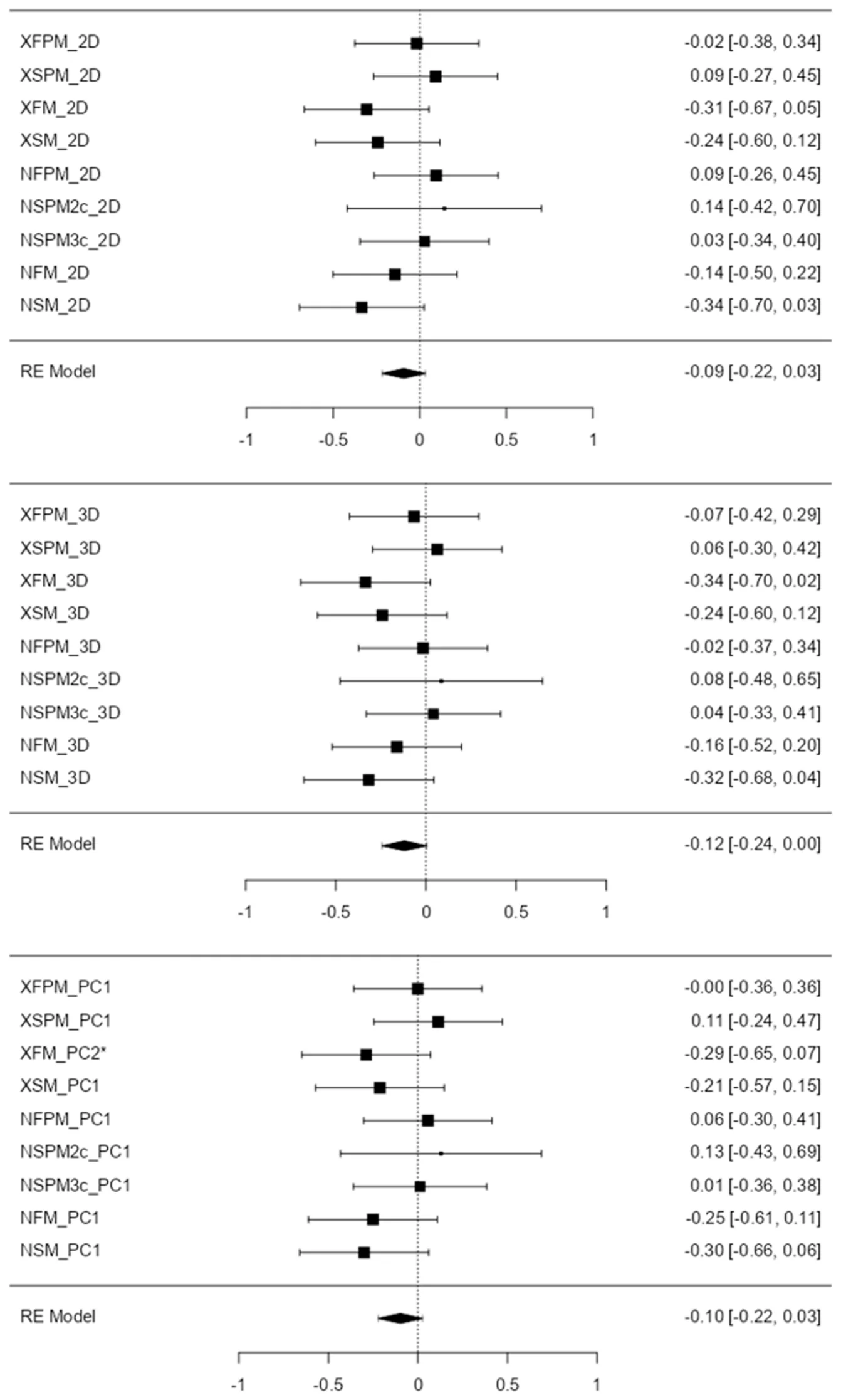
Forest plot showing pooled sex differences for principal component-derived size variables, 2D centroid size, and 3D centroid size across all tooth types. * For the maxillary first molar, centroid size variables loaded predominantly on PC2 and therefore PC2 was used for comparison. Squares indicate individual effect estimates and their weights; horizontal lines represent 95% confidence intervals. The diamond denotes the pooled random-effects estimate, and its width corresponds to the 95% confidence interval.

**Figure 3:**
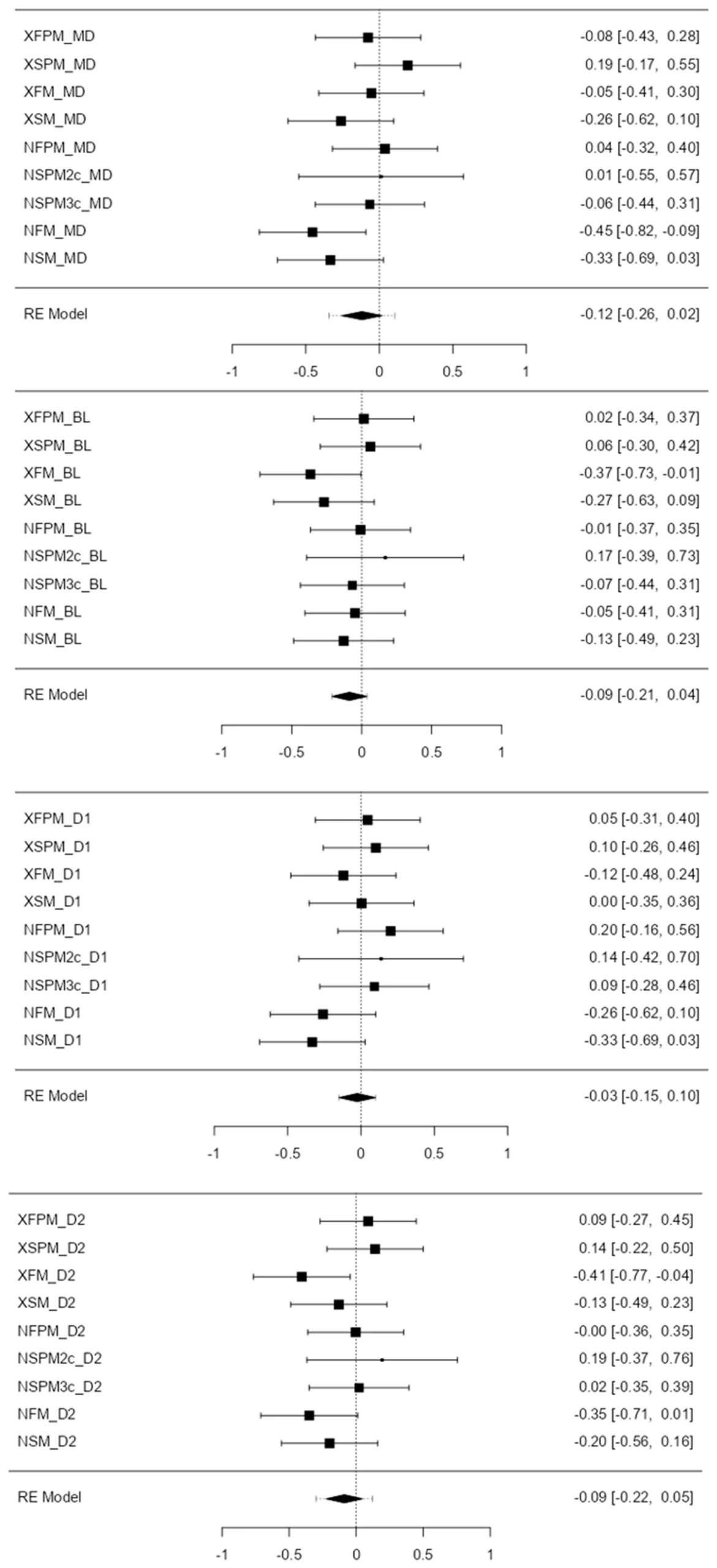
Forest plot showing pooled sex differences for conventional odontometric measurements (mesiodistal, buccolingual, diagonal 1, and diagonal 2 dimensions) across all tooth types. Squares indicate individual effect estimates and their weights; horizontal lines represent 95% confidence intervals. The diamond denotes the pooled random-effects estimate, and its width corresponds to the 95% confidence interval.

The pooled meta-analysis results are presented in Table 5, with the corresponding forest plots shown in Figure 2 and Figure 3. Across all measurement approaches, the pooled effect sizes were small and non-significant, indicating substantial overlap in tooth size between males and females.

For the multidimensional size variables, the pooled effect size for the principal component-derived measurements was −0.098 (95% CI: −0.222 to 0.026; *p* = 0.123), while the pooled effect sizes for the 2D and 3D centroid measurements were −0.092 (95% CI: −0.217 to 0.032; *p* = 0.146) and −0.120 (95% CI: −0.244 to 0.005; *p* = 0.059), respectively. None of these pooled estimates demonstrated statistically significant sexual dimorphism.

Similarly, the conventional odontometric measurements demonstrated small pooled effect sizes. The pooled Cohen’s d values were −0.118 (95% CI: −0.256 to 0.020; *p* = 0.093) for mesiodistal dimensions, −0.088 (95% CI: −0.212 to 0.037; *p* = 0.167) for buccolingual dimensions, −0.025 (95% CI: −0.149 to 0.099; *p* = 0.690) for diagonal 1 measurements, and −0.088 (95% CI: −0.224 to 0.048; *p* = 0.204) for diagonal 2 measurements. Among all methods evaluated, diagonal 1 demonstrated the smallest pooled effect size, whereas mesiodistal measurements showed the largest, but neither reached statistical significance.

The heterogeneity across tooth types was negligible for most measurement methods. No heterogeneity was observed for the principal component-derived size variables, 2D centroid size, 3D centroid size, buccolingual dimensions, and diagonal 1 measurements (I^2^ = 0%). Low heterogeneity was observed for mesiodistal dimensions (I^2^ = 18.2%) and diagonal 2 measurements (I^2^ = 15.4%), indicating consistent findings across the evaluated tooth types.

Equivalence testing demonstrated statistically significant equivalence for all measurement approaches (all TOST *p* < 0.001). The pooled confidence intervals for the principal component-derived variables, centroid size measurements, and conventional odontometric dimensions remained well within the predefined equivalence bounds of Cohen’s d = ±0.5. These findings indicate that the observed differences between males and females were not only statistically non-significant but also sufficiently small to be considered practically equivalent.

The meta-analysis provides strong evidence that tooth size, when assessed using conventional linear measurements, centroid-based geometric morphometric measures, or principal component-derived multidimensional constructs, demonstrates practical equivalence between sexes.

Overall, our results consistently demonstrated that conventional odontometric measurements, centroid size measurements, and principal component-derived variables captured a common underlying size construct. Individual dimensions showed varying contributions to this construct; however, none of the integrated multidimensional size variables demonstrated significant sexual dimorphism. The pooled effect sizes across all analytical approaches were small and demonstrated substantial overlap between males and females. The findings support practical equivalence within the specified equivalence bounds rather than complete biological equivalence.

## 4. Discussion

The present study evaluated tooth size using three conceptually different approaches. The first level is constituted by the mesiodistal, buccolingual and diagonal measurements, which constitute the conventional odontometric methods. Centroid size provides a geometric estimate of overall size derived from the spatial arrangement of all landmarks and is independent of any single dimension. The third level is represented by the use of principal component analysis which statistically combined multiple correlated measurements into a composite size variable. Therefore, centroid size and PCA-derived variables do not replace conventional measurements but provide complementary multidimensional assessments that may better represent the complex three-dimensional nature of tooth morphology. The strong correlations observed between centroid size and PCA-derived variables in the present study indicate that both approaches captured a common underlying size construct. However, centroid size derives directly from geometric landmark configurations, whereas PCA derives a latent size variable from the covariance structure among multiple measurements. This distinction is important because the two methods represent different analytical pathways toward evaluating overall tooth size while overcoming the limitations associated with isolated linear measurements.

Our study differs from previous odontometric studies in two ways. Previous studies relied on null-hypothesis testing of mesiodistal, buccolingual, or diagonal measurements to evaluate sexual dimorphisms; however, we used equivalence testing and meta-analysis to determine similarities between the sexes. Secondly, to the best of our knowledge, this is the first study to combine multiple linear measurements and geometric morphometric size variables as a “size construct” for evaluating tooth size. Our study demonstrated that isolated dimensions occasionally showed statistically significant differences between males and females. Equivalence testing demonstrated practical equivalence for four tooth categories, whereas the remaining categories did not provide sufficient evidence to establish equivalence within the predefined bounds. Nevertheless, none of the evaluated multidimensional size variables demonstrated statistically significant sex differences, and the observed effect sizes were generally small, suggesting substantial overlap in overall tooth size between males and females. Within the limits of the present study, multidimensional assessments of postcanine tooth size demonstrated substantial overlap between males and females. Although some tooth categories met predefined equivalence criteria, the findings should be interpreted as evidence of practical similarity rather than definitive biological equivalence. Failure to demonstrate equivalence should not be interpreted as evidence of a meaningful difference.

Sexual dimorphism in tooth dimensions has long been utilized in forensic anthropology and forensic odontology because teeth are among the most durable structures of the human body [30,31,32]. Traditional odontometric studies have reported varying degrees of sexual dimorphism in mesiodistal and buccolingual dimensions, particularly in canines and molars [33,34,35]. However, findings have remained inconsistent across populations. A recent systematic review of CBCT-based odontometric studies by Ajmal et al. reported considerable variation in the magnitude and pattern of sexual dimorphism among different populations, suggesting that sex-related differences in tooth dimensions are influenced by both biological and methodological factors [36]. They reported that several studies included in the review failed to identify significant sex differences despite using advanced three-dimensional imaging methods.

Conventional odontometric analysis evaluates only a few linear dimensions from one of the five aspects of a tooth which are inherently three-dimensional structures. Mesiodistal, buccolingual, and diagonal dimensions capture only limited aspects of crown dimensions and may not adequately characterize overall crown size [5,37,38,39]. Bernal (2007) compared the traditional and geometric morphometric approaches in human molars, and demonstrated that landmark-based methods provide a more comprehensive assessment of biological form by distinguishing size-related variation from shape-related variation [12]. This is particularly important when interpreting the sexual dimorphism of three-dimensional structures as differences observed in one dimension may not necessarily reflect differences in overall tooth size.

The principal component analysis performed in the present study demonstrated that the combination of the conventional odontometric dimensions and centroid size measurements largely represented a common underlying size construct of the teeth. For eight of the nine tooth categories, a single principal component explained between 79.1% and 91.3% of the total variance. A single principal component, accounting for a substantial proportion of the total variance and showing very strong correlations with both 2D and 3D centroid sizes, indicates that mesiodistal, buccolingual, diagonal, and centroid measurements represent different expressions of the same underlying multidimensional tooth-size variable rather than independent morphological traits. As many studies analyze isolated measurements to demonstrate sexual dimorphism, the present results suggest that these measurements should instead be considered components of a broader, multidimensional size phenotype.

An important observation was the consistently high loadings of centroid-size variables across all tooth categories. In every tooth type, the two-dimensional centroid size exhibited the highest loading, while the three-dimensional centroid size exhibited either the highest or second-highest loading. These findings indicate that centroid size captures the common size construct more effectively than any individual linear measurement. Unlike conventional odontometric parameters, centroid size incorporates information from all landmarks simultaneously and therefore provides a holistic representation of tooth size. Similar observations have been reported in geometric morphometric studies where centroid size has been shown to represent global size variation while minimizing the influence of individual dimensional fluctuations. Natarajan et al. demonstrated that centroid size represented a stable measure of overall tooth size in mandibular molars, whereas shape variables accounted for most sex-related variation [25,40,41].

The correlation analysis further strengthened this interpretation. The principal component-derived size variables demonstrated extremely strong correlations with both two-dimensional and three-dimensional centroid sizes, with correlation coefficients ranging from 0.945 to 0.994. The consistency of these findings across all tooth categories suggests that centroid size may serve as a robust surrogate measure of multidimensional tooth size. From a methodological perspective, this observation supports using centroid size as a biologically meaningful and statistically efficient measure in future investigations of tooth size variation.

The maxillary first molar demonstrated a unique pattern compared with the other teeth. Two principal components were required to explain the observed variation, with PC1 accounting for 48.1% and PC2 for 34.1%. The centroid variables loaded predominantly on PC2 rather than PC1. This suggests that the maxillary first molar possesses a more complex size architecture than the remaining tooth types. This is well supported by research by Morita et al. (2016), which demonstrated that the maxillary first molar is phenotypically distinct among the maxillary molars [42]. As the maxillary first molar is morphologically the largest and most anatomically complex posterior tooth with multiple cusps, triangular ridges, grooves and fossae, single dimensions may not represent the crown size. Similar observations have been reported in recent geometric morphometric studies, in which molars frequently exhibit more complex patterns of variation than premolars. Bianchi et al. (2023) similarly reported that premolar-derived principal components exhibited stronger discriminatory performance between sexes, than molar-derived components, suggesting that premolars may represent more homogeneous morphological units, whereas molars contain additional sources of variation that are not fully captured by a single latent variable [19].

The present study found no significant sex differences in the integrated size variables derived from PCA. Although isolated odontometric dimensions occasionally demonstrated statistically significant differences, none of the principal component-derived size variables showed significant differences between males and females. Effect sizes were uniformly small, ranging from 0.012 to 0.300. These findings suggest that apparent differences observed in individual dimensions may be compensated by reciprocal variation in other dimensions. In other words, a tooth that appears larger in one measurement may simultaneously be smaller in another measurement, resulting in little overall difference in multidimensional size.

The present findings also have implications beyond morphometric research. In forensic odontology, odontometric measurements are frequently incorporated into sex estimation protocols because teeth remain among the most durable biological structures recovered from human remains. However, recent systematic reviews have highlighted the considerable heterogeneity with moderate diagnostic performance of conventional linear odontometric models, concluding that linear crown dimensions alone should not be considered definitive for sex estimation [21,22]. Our findings provide a possible explanation for these observations by demonstrating that statistically significant differences in isolated mesiodistal or buccolingual dimensions do not necessarily translate into meaningful differences in overall multidimensional tooth size. This suggests that future models may benefit from integrating three-dimensional geometric morphometric descriptors with conventional odontometric variables rather than relying exclusively on individual linear measurements.

This phenomenon provides a plausible explanation for the inconsistent findings reported in the literature involving odontometric measurements. While studies conducted in Australian adolescents, Brazilian children, and Portuguese populations have reported sexual dimorphism across most posterior teeth [5,37,43], other investigations from Iranian and South Indian populations have demonstrated sexual dimorphism only in selected teeth or dimensions, with several teeth/dimensions showing no significant sex differences [44,45]. At the other end of the spectrum, studies from Nigerian populations have reported an absence of significant sexual dimorphism in posterior tooth dimensions altogether [46]. Such variability suggests that conclusions regarding odontometric sexual dimorphism may be influenced by the specific teeth examined, population characteristics, and, importantly, the methodology involving the dimensions used to quantify tooth size.

**Figure 4:**
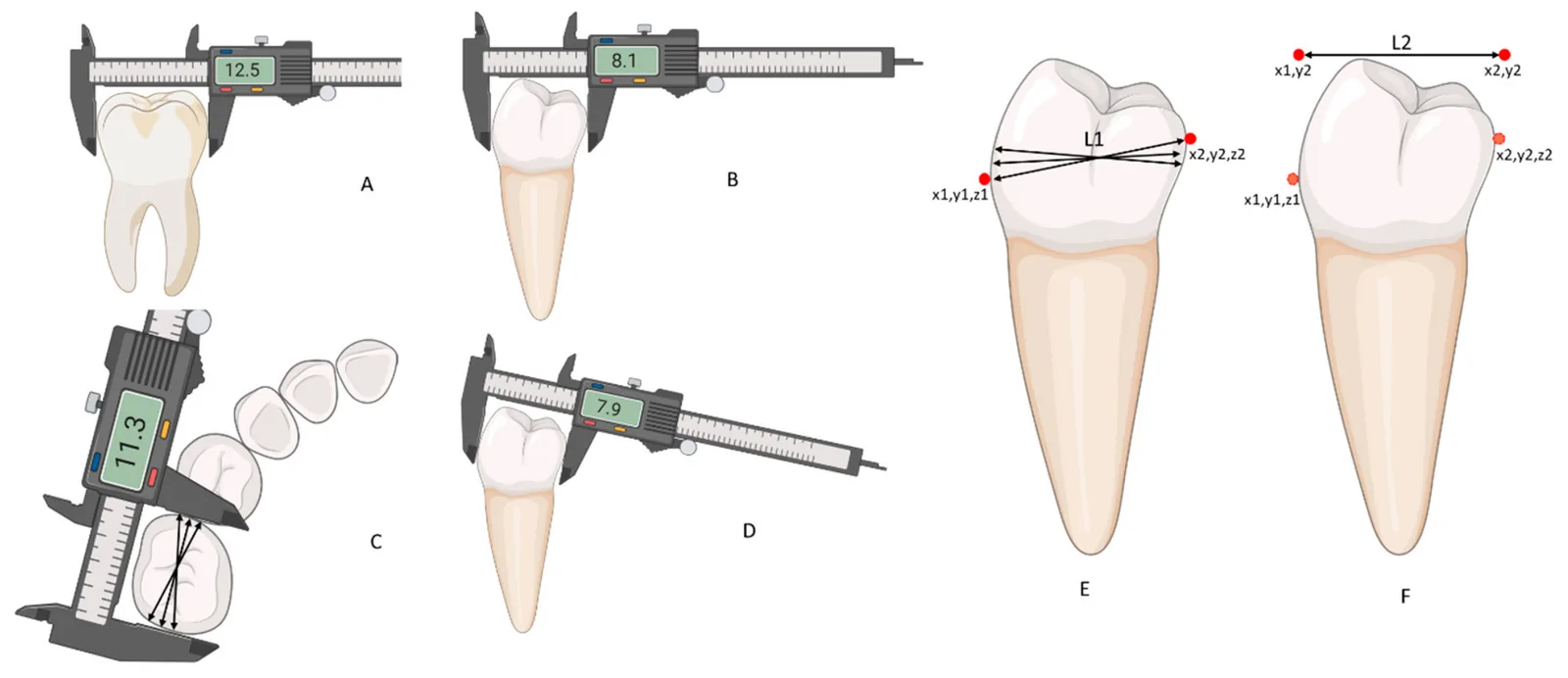
(**A**) represents the mesiodistal measurement of a molar taken using a vernier caliper. (**B**) denotes the buccolingual measurement of a premolar taken with the same instrument. (**C**,**D**) highlight potential measurement variation due to changes in the vernier caliper’s angulation during use on occlusal and proximal surfaces. (**E**) depicts the locations of the crests of curvature on the buccal and lingual contours of the tooth, represented by two red dots, with their coordinates plotted in three-dimensional space (x, y, z). L1 shows the variable distances measured directly by the vernier caliper. (**F**) illustrates the calculation of the corrected linear distance (L2) between the two crests of curvature after eliminating the *z*-axis coordinates. This is computed using the formula L2 = Sqrt [(x_2_ − x_1_)^2^ + (y_2_ − y_1_)^2^].

The angulation, placement, and orientation of measuring instruments can influence conventional odontometric measurements. Even minor deviations in vernier caliper positioning may alter mesiodistal or buccolingual dimensions (Figure 4). In contrast, landmark-based measurements use Cartesian coordinates and Euclidean distance calculations, making them independent of instrument placement and providing greater precision and reproducibility.

The concept of dimensional compensation is particularly relevant when evaluating three-dimensional biological structures. Conventional odontometric measurements reduce a complex three-dimensional morphology into a limited number of linear dimensions. As a result, isolated measurements may exaggerate localized differences while failing to capture the overall size of the structure. Geometric morphometric approaches overcome this limitation by simultaneously incorporating information from multiple anatomical locations. Garizoain et al. emphasized that biological morphology should be interpreted as an integrated combination of size and shape rather than as isolated linear measurements [33]. Similarly, recent geometric morphometric studies have demonstrated that shape variation often contains more sexually dimorphic information than size variation alone [25].

The present study differs from other odontometric studies in that our null hypothesis was that size is equivalent between sexes. The lack of a significant difference does not necessarily mean that the two sizes are similar. An important observation was that several tooth categories showed non-significant results in conventional hypothesis tests while failing to meet the criteria for statistical equivalence. This outcome represents a zone of statistical indefinity, where the available evidence is insufficient to conclude either that there is a meaningful difference or that there is practical equivalence between males and females. In such situations, the confidence intervals are too wide to exclude differences exceeding the predefined equivalence bounds, suggesting that uncertainty remains about the true magnitude of the sex effect. Interestingly, although equivalence could not be established for several individual tooth categories, the pooled meta-analytic results demonstrated significant equivalence across all measurement approaches, including principal component scores, centroid sizes, and conventional odontometric dimensions. This apparent discrepancy arises because meta-analysis combines information across multiple teeth, thereby increasing statistical precision and reducing uncertainty around the pooled effect estimate. Thus, while individual teeth may not provide sufficient evidence to establish equivalence on their own, the collective evidence from the dentition supports the conclusion that overall differences in tooth size between males and females are small and fall within the predefined equivalence margins. These findings highlight an important distinction between the absence of evidence and evidence of equivalence. Individual tooth analyses frequently lacked sufficient precision to conclusively demonstrate equivalence, whereas the pooled meta-analytic approach provided robust evidence that any sex-related differences in tooth size were of negligible practical importance. To the best of our knowledge, the application of formal equivalence testing to odontometric measurements has not been reported previously. The present study therefore extends the methodological framework of odontometric research by evaluating not only whether males and females differ, but also whether their tooth sizes are statistically equivalent within predefined bounds of effect size.

Our recent investigations demonstrated that shape-based geometric morphometric variables could classify sex with high accuracy using machine-learning approaches, despite minimal differences in centroid size [41]. Similarly, we have reported that premolar and molar morphology show stronger discriminatory power of shape variables than the size variables [24,25]. Collectively, these findings suggest that sexual dimorphism in postcanine teeth may be expressed predominantly through shape rather than overall size. Therefore, future investigations may benefit from focusing on landmark configurations and shape variables rather than relying solely on conventional size measurements.

In the present study, the mesiodistal, buccolingual, and diagonal widths constitutes the conventional measure and centroid size represents a geometric morphometric measure of overall size derived from the spatial configuration of landmarks. The principal component-derived size variables represent statistical constructs that integrate information from multiple correlated measurements into a single multidimensional size variable. Centroid size should not be interpreted as a complete geometric morphometric analysis. Rather, it represents a landmark-derived measure of overall size obtained from geometric morphometric data [47]. In contrast, shape analysis in geometric morphometrics requires Procrustes superimposition, which removes variation attributable to size, position, and orientation to isolate shape differences [48]. The present study focused exclusively on size-related variation and therefore did not evaluate Procrustes-derived shape variables directly.

The clinical relevance of the present study lies in the demonstration of the fact that overall tooth size is best understood as a multidimensional construct rather than as a collection of isolated linear measurements. Although statistically significant differences may occasionally be observed in individual odontometric dimensions, these differences do not necessarily translate into meaningful differences in overall tooth size. The strong agreement observed between conventional measurements, centroid size, and principal component-derived variables suggests that multidimensional approaches provide a comprehensive representation of tooth size while accounting for the complex three-dimensional morphology of dental structures. We must remain cognizant of these findings in designing applications for digital dentistry, orthodontic diagnosis, restorative and prosthodontic treatment planning, dental anthropology, and future artificial intelligence-based morphometric analyses, where accurate representation of overall tooth morphology is important [49,50].

The strengths of the present study include the use of three-dimensional landmark coordinates, the evaluation of multiple tooth types, the integration of conventional and geometric morphometric measurements, the application of PCA-derived size variables, and the incorporation of both equivalence testing and meta-analysis. These approaches allowed tooth size to be examined as a multidimensional biological construct rather than as a collection of isolated measurements.

This study has a few limitations related to its design and interpretation. The study was conducted in a single population, and the findings may not be directly generalizable to other ethnic groups. Additionally, the investigation focused primarily on size-related variables and did not independently evaluate shape variation. Future studies incorporating Procrustes-based geometric morphometric analyses and machine learning classification methods may provide further insight into the relative contributions of size and shape to sexual dimorphism. The meta-analyses were performed to provide an overall summary of effect sizes across tooth types within the same individuals. Thus, complete statistical independence among effect sizes cannot be assumed and therefore the pooled estimates should be interpreted as descriptive summaries of the observed patterns rather than independent confirmatory evidence. Future studies employing multilevel meta-analytic or mixed-effects modeling approaches may provide a more rigorous evaluation method for accounting for within-subject correlations among tooth-specific effect sizes. The sample size calculation was based on previously reported conventional odontometric measurements rather than PCA-derived variables or equivalence testing. Consequently, while the study was adequately powered for the primary comparative analyses, the equivalence results should be interpreted within the context of the available sample size and predefined equivalence margins. Future investigations specifically powered for multidimensional and equivalence-based analyses are warranted.

## 5. Conclusions

In conclusion, the present study demonstrates that conventional odontometric measurements, centroid size variables, and PCA-derived multidimensional size measures largely represent the same underlying size construct. Although isolated dimensions occasionally demonstrated statistically significant sex differences, multidimensional analyses consistently revealed substantial overlap. While formal equivalence was demonstrated for several tooth categories, the evidence does not support universal equivalence across all postcanine teeth. Rather, the results suggest that sex-related differences in overall tooth size are limited and smaller than traditionally implied by isolated odontometric measurements. Centroid size emerged as the variable most strongly associated with the common size construct and may therefore represent the most biologically meaningful measure of tooth size in future odontometric and forensic investigations. The findings suggest that multidimensional assessment methods may provide a more comprehensive framework for evaluating tooth size in research and clinical applications involving complex three-dimensional dental morphology.

## Figures and Tables

**Table 1:** Principal component loadings and variance explained.

Tooth	MD	BL	D1	D2	2D Centroid	3D Centroid	Variance Explained (%)
Maxillary First Premolar (XFPM)	0.798	0.902	0.938	0.926	0.984	0.973	85.0
Maxillary Second Premolar (XSPM)	0.835	0.919	0.950	0.955	0.989	0.986	88.4
Maxillary First Molar (XFM)	0.812	0.832	0.843	0.847	0.967 *	0.963 *	82.2 *
Maxillary Second Molar (XSM)	0.917	0.755	0.862	0.880	0.985	0.945	79.9
Mandibular First Premolar (NFPM)	0.752	0.853	0.843	0.917	0.983	0.969	79.1
Mandibular Second Premolar two-cusp type (NSPM2c)	0.919	0.925	0.939	0.960	0.994	0.992	91.3
Mandibular Second Premolar three-cusp type (NSPM3c)	0.841	0.862	0.924	0.873	0.992	0.986	83.7
Mandibular First Molar (NFM)	0.874	0.833	0.950	0.935	0.985	0.985	86.2
Mandibular Second Molar (NSM)	0.940	0.788	0.924	0.935	0.985	0.972	85.8

* Two components were retained for the maxillary first molar. PC1 explained 48.1% and PC2 explained 34.1% of the variance (cumulative variance = 82.2%). Centroid variables loaded predominantly on PC2.

**Table 2:** Correlation of principal component-derived size variables with 2D and 3D centroid sizes.

Tooth	Principal Component	2D Centroid r ^ⴃ^	3D Centroid r ^ⴃ^
Maxillary First Premolar (XFPM)	PC1	0.984	0.973
Maxillary Second Premolar (XSPM)	PC1	0.989	0.986
Maxillary First Molar (XFM) *	PC2	0.967	0.963
Maxillary Second Molar (XSM)	PC1	0.985	0.945
Mandibular First Premolar (NFPM)	PC1	0.983	0.969
Mandibular Second Premolar two-cusp type (NSPM2c)	PC1	0.994	0.992
Mandibular Second Premolar three-cusp type (NSPM3c)	PC1	0.992	0.986
Mandibular First Molar (NFM)	PC1	0.985	0.985
Mandibular Second Molar (NSM)	PC1	0.985	0.972

* For the maxillary first molar, the centroid variables loaded predominantly on PC2 rather than PC1; therefore, correlations are reported with PC2. ^ⴃ^: All Pearson correlation coefficients were statistically significant (*p* < 0.001).

**Table 3:** Comparison of principal component-derived size variables between males and females.

Tooth Type	Principal Component	Cohen’s d	*p* Value
Maxillary First Premolar (XFPM)	PC1	−0.001	0.997
Maxillary Second Premolar (XSPM)	PC1	0.113	0.536
Maxillary First Molar (XFM)	PC2 *	−0.289	0.116
Maxillary Second Molar (XSM)	PC1	−0.213	0.246
Mandibular First Premolar (NFPM)	PC1	0.056	0.760
Mandibular Second Premolar—two-cusp (NSPM2c)	PC1	0.129	0.658
Mandibular Second Premolar—three-cusp (NSPM3c)	PC1	0.012	0.950
Mandibular First Molar (NFM)	PC1	−0.252	0.171
Mandibular Second Molar (NSM)	PC1	−0.300	0.103

* For the maxillary first molar, centroid size variables loaded predominantly on PC2 and therefore PC2 was used as the integrated size variable for sex comparison.

**Table 4:** Equivalence Testing of Principal Component–Derived Tooth Size Variables Using the Two One-Sided Tests (TOST) Procedure.

Tooth Type	Component	NHST *p* Value	TOST Lower *p* Value	TOST Upper *p* Value	Conclusion
Maxillary First Premolar (XFPM)	PC1	0.997	0.004	0.004	Equivalent
Maxillary Second Premolar (XSPM)	PC1	0.536	<0.001	0.019	Equivalent
Maxillary First Molar (XFM)	PC1	0.077	0.164	<0.001	Failed Lower Bound
Maxillary First Molar (XFM) *	PC2	0.117	0.121	<0.001	Failed Lower Bound
Maxillary Second Molar (XSM)	PC1	0.246	0.059	<0.001	Failed Lower Bound
Mandibular First Premolar (NFPM)	PC1	0.760	0.001	0.009	Equivalent
Mandibular Second Premolar—Two-Cusp Type (NSPM2c)	PC1	0.625	0.011	0.085	Failed Upper Bound
Mandibular Second Premolar—Three-Cusp Type (NSPM3c)	PC1	0.951	0.005	0.007	Equivalent
Mandibular First Molar (NFM)	PC1	0.171	0.087	<0.001	Failed Lower Bound
Mandibular Second Molar (NSM)	PC1	0.103	0.133	<0.001	Failed Lower Bound

* For the maxillary first molar, centroid size variables loaded predominantly on PC2 and therefore PC2 was used for comparison.

**Table 5:** Meta-analysis summary.

Method	Pooled Cohen’s d (95% CI)	*p* Value	I^2^ (%)	TOST *p* Value
PC Component	−0.098 (−0.222 to 0.026)	0.123	0.0	<0.001
2D Centroid	−0.092 (−0.217 to 0.032)	0.146	0.0	<0.001
3D Centroid	−0.120 (−0.244 to 0.005)	0.059	0.0	<0.001
MD	−0.118 (−0.256 to 0.020)	0.093	18.2	<0.001
BL	−0.088 (−0.212 to 0.037)	0.167	0.0	<0.001
D1	−0.025 (−0.149 to 0.099)	0.690	0.0	<0.001
D2	−0.088 (−0.224 to 0.048)	0.204	15.4	<0.001

## Data Availability

The datasets used and/or analyzed during the current study are available from the corresponding author on request.

## Abbreviations

The following abbreviations are used in this manuscript:

XFPM	Maxillary First Premolar
XSPM	Maxillary Second Premolar
XFM	Maxillary First Molar
XSM	Maxillary Second Molar
NFPM	Mandibular First Premolar
NSPM2c	Mandibular Second Premolar—2 cusp type
NSPM3c	Mandibular Second Premolar—3 cusp type
NFM	Mandibular First Molar
NSM	Mandibular Second Molar
MD	Mesiodistal
BL	Buccolingual
D1	Mesiobuccal to Distolingual Diagonal
D2	Distobuccal to Mesiolingual Diagonal
PC	Principal Component
